# Posterolateral route for a midbrain cavernous malformation reaching the anterior surface of the brainstem

**DOI:** 10.3171/2019.7.FocusVid.19162

**Published:** 2019-07-01

**Authors:** Michel W. Bojanowski, Gunness V. R. Nitish, Gilles El Hage, Kim Lalonde, Chiraz Chaalala, Thomas Robert

**Affiliations:** 1 Division of Neurosurgery, Department of Surgery, Centre Hospitalier de L’Université de Montreal, Quebec, Canada; and; 2 Department of Neurosurgery, Regional Hospital of Lugano, Lugano, Switzerland

**Keywords:** cavernous malformation, surgical resection, brainstem, supracerebellar infratentorial, video

## Abstract

Cavernous malformations in the midbrain can be accessed via several safe entry zones. The accepted rule of thumb is to enter at the point where the lesion is visible at the surface of the brainstem to pass through as little normal brain tissue as possible. However, in some cases, in order to avoid critical neural structures, this rule may not apply. A different safe entry zone can be chosen. Our video presents a case of a ruptured cavernous malformation in the midbrain reaching its anterior surface which was successfully resected via a posterolateral route using the supracerebellar infratentorial approach.

The video can be found here: https://youtu.be/7kt-OQuBmz0.

## Transcript

My name is Michel Bojanowski and herein I present a case of a ruptured midbrain cavernoma reaching the surface of the midbrain anteriorly, which we approached posteriorly in order to minimize invasiveness.

This 30-year-old female with no previous medical history presented with a rapid onset of acute dysarthria with left oculomotor paresis. MRI revealed an anterior midbrain ruptured cavernoma. Since, when seen, the patient was stable, we opted for a conservative approach.

However, 1 month later she experienced a new bleed with severe right hemiparesis, right hypoesthesia, diplopia, and severe dysarthria.

The hemorrhage reached the surface of the midbrain anteriorly and extended posteriorly without reaching the surface of the posterior safe entry zone. Because the cavernoma had already bled twice, we proposed its surgical resection (Garcia et al., 2015).

Two approaches may be considered. One approach is via an orbitozygomatic route to reach the anterior midbrain to which surface the hematoma extends (Mascitelli et al., 2019). However, this approach requires working in close proximity to the perforators of the basilar artery, and to the motor pathway in the cerebral peduncle.

In our case, we preferred the shorter posterior route via a supracerebellar approach to use the lateral mesencephalic safe entry zone (Abla et al., 2010; Ding et al., 2017; Giliberto et al., 2010).

In park bench position, with the left side up, head flexed and elevated, a left paramedian skin incision is made to expose the occipital bone. A suboccipital craniotomy is performed extending upward to expose the inferior portion of the transverse sinus, and laterally to expose the medial border of the sigmoid sinus.

The dura is opened in a triangular shape with the base at the transverse sinus.

Under the surgical microscope, using the infratentorial supracerebellar approach, the tentorium is followed anteriorly to expose the cerebellomesencephalic cistern.

The cistern is opened using a beaver blade. Here, we can clearly see the fourth cranial nerve between the duplicated superior cerebellar artery. The arachnoid is further incised. A very mild yellowish discoloration is barely perceptible at the level of the lateral mesencephalic fissure. The lateral mesencephalic vein is clearly identifiable and will be preserved throughout the entire procedure. The neuronavigator confirms the location of the hematoma below. A small incision of the pia is made using the beaver blade, and we proceed to dissect the superficial layers of the brainstem to reach the lesion, without using any coagulation.

The soft part of the hematoma is first suctioned out and the more solid part is then removed using tweezers. We proceed with extracapsular dissection of the cavernoma, which can be removed by gradually stretching the working window as we work. Microscissors are used to detach the cavernoma from the surrounding tissue. Using tweezers and by going along with the natural pulsation of the brain while respecting the viscoelasticity of the tissues, the cavernoma is extracted in large chunks. The bed of the cavernoma is explored to ensure complete resection. Hemostasis is ensured.

Following watertight closure of the dura and repositioning of the bone flap, closure is done in the usual fashion.

In a few days, the patient improved significantly neurologically, without any new deficits.

MRI showed complete resection of the lesion.

### Time points

1:24 Justification choice of approach

2:28 Exposure of the cerebellomesencephalic cistern

3:17 Identification of the lateral mesencephalic vein

3:25 Use of neuronavigation to localize the lesion

3:30 Exposure of the cavernoma in the brainstem

3:54 Intralesional resection of the cavernous malformation

4:22 Extracapsular dissection

5:05 Resection of the cavernous malformation
